# Printed Ag Mesh Electrodes with Enhanced Adhesion on Diverse Substrates for Transparent Heater Applications

**DOI:** 10.3390/nano15211681

**Published:** 2025-11-05

**Authors:** Han-Jung Kim, Se Yong Park, Jeongmin Park, Yohan Ko, Changjoo Shin, Dong-Woo Man, Yoonkap Kim

**Affiliations:** 1 IT Materials & Components Research Center, Gumi Electronics & Information Technology Research Institute (GERI), Cheomdangieop1-ro 17, Sandong-eup, Gumi 39171, Republic of Korea; hjkim0321@geri.re.kr (H.-J.K.); psy881024@geri.re.kr (S.Y.P.); gumi00064@geri.re.kr (J.P.); yohan@geri.re.kr (Y.K.); 2 Maritime ICT & Mobility Research Department, Korea Institute of Ocean Science & Technology (KIOST), 385 Haeyang-ro, Yeongdo-gu, Busan 49111, Republic of Korea; cjshin@kiost.ac.kr

**Keywords:** inkjet printing, Ag mesh, flexible electrode, transparent heater, printed electronics

## Abstract

Digital printing technologies—including inkjet printing, aerosol jet printing, and electrohydrodynamic jet printing—have emerged as promising strategies for next-generation electronic devices. However, the weak adhesion between printed electrodes and substrates can lead to electrode delamination, thereby compromising device reliability and lifetime. In this study, a dielectric interlayer was introduced to improve the adhesion of silver (Ag) mesh electrodes on glass, polyethersulfone film, and polyimide film substrates. The optimized electrode on PES film achieved an optical transmittance of 83% at 550 nm and line resistance of 0.3 Ω, confirming its suitability as a transparent electrode. The incorporation of the interlayer also enhanced the adhesion and mechanical flexibility across all substrates. Moreover, the printed electrodes exhibited uniform surface heating under an applied bias (≤DC 3 V), and their feasibility as low-power flexible transparent heaters was experimentally demonstrated. These findings present a simple and effective printing strategy for fabricating robust and multifunctional electrodes, offering enormous potential for the realization of future flexible and transparent electronic systems.

## 1. Introduction

Printed electronics have emerged as promising platforms for next-generation electronic devices, owing to their inherent low cost, large-area scalability, environmental sustainability advantages, and excellent compatibility with flexible substrates [1,2,3,4,5,6,7,8,9,10]. Despite these merits, weak interfacial adhesion between printed electrodes and substrates remains a critical bottleneck [11,12,13,14,15,16,17,18]. Such poor adhesion can result in electrode delamination under external mechanical stress, ultimately leading to severe performance degradation and shortened device lifetimes.

To overcome this limitation, the incorporation of adhesion layers has been extensively investigated in recent years [14,19,20,21]. However, conventional adhesion layers generally suffer from two major drawbacks—that is, a reduction in optical transmittance and an increase in process complexity. These limitations hinder their practical deployment in flexible and transparent electronic devices.

In this study, we introduced a novel electrode architecture in which a dielectric material—functioning as an adhesion-promoting interlayer—was accurately printed in a mesh pattern, followed by the subsequent printing of conductive silver (Ag) ink. Through this sequential printing strategy, Ag mesh electrodes could be fabricated on a variety of substrates, including glass, polyethersulfone (PES) films, and polyimide (PI) films. This method effectively reinforced the adhesion and mechanical flexibility of the electrodes while preserving their intrinsic optical transparency and electrical conductivity.

The resulting Ag mesh electrodes exhibited outstanding optical and electrical properties, along with robust adhesion and durability under repeated mechanical deformation. Moreover, they successfully operated as transparent heaters, generating uniform heat at low voltages while maintaining stable performance under bending and twisting conditions. These findings demonstrate that adhesion-enhanced printed electrodes with dielectric interlayers hold considerable promise as key enabling components for future flexible and transparent electronic systems.

## 2. Materials and Methods

### 2.1. Fabrication of Heaters via Printing Processes

Printed heaters were fabricated via inkjet printing using a DragonFly IV 3D printer (Nano Dimension, Israel) [14,22,23,24]. Ag nanoparticle ink (CI-90072, Nano Dimension) was printed in a mesh pattern onto various substrates, including glass, PES film, and PI film. Prior to Ag ink printing, a dielectric ink (DI-1092, Nano Dimension) was pre-patterned onto the substrates in an identical mesh pattern to improve the interfacial adhesion between the Ag structures and substrates. During the printing process, the substrate temperature was maintained at 160 °C to facilitate the in situ sintering of the printed inks. Following the printing step, the dielectric ink was rapidly cured using ultraviolet (UV, 395 nm) irradiation, whereas the Ag conductive ink was cured using infrared (IR, 815 nm) irradiation. This combined curing process ensured sufficient structural integrity and electrical functionality of the printed heater devices.

### 2.2. Structural, Optical, and Electrical Evaluation of Inkjet-Printed Heaters

The microstructure of the Ag mesh-based heaters was characterized using an optical microscopy (OM; Olympus, BM51M) and field-emission scanning electron microscopy (FE-SEM; JEOL, JSM-7610F). The optical transmittance of the fabricated heaters was measured using a UV–Vis spectrophotometer (Agilent Cary 5000, CA, USA). Owing to the non-continuous nature of the Ag micro-mesh structures, the electrical performance was evaluated by measuring the line resistance between the two ends of the heater using a digital multimeter (FLUKE 117, Everett, USA), instead of conventional sheet resistance measurements [14].

### 2.3. Adhesion Test of Inkjet-Printed Ag Mesh Structures on Various Substrates

Adhesion of the inkjet-printed Ag mesh structures to the substrates was evaluated using two methods. First, samples were immersed in deionized (DI) water and subjected to ultrasonication, and the change in electrical resistance was recorded [25,26]. Second, a cellophane adhesive tape (3M 610, MN, USA) was applied to the electrode surface and then peeled off, followed by resistance measurements to assess the delamination of the printed electrodes [14,27]. The change in resistance during the adhesion and mechanical flexibility tests was recorded using a custom-built measurement setup capable of detecting variations in the milliohm range.

### 2.4. Performance Evaluation of the Printed Heater

The surface temperature of the printed heater was evaluated using both contact and non-contact methods. For the contact-based measurement, a type-K thermocouple (RKC Instrument INC. ST-50) was attached directly to the non-printed side of the substrate—that is, the side opposite the inkjet-printed Ag mesh—using thermally conductive adhesive tape to ensure stable thermal contact. The temperature data were recorded in real time using a digital data acquisition system (Graphtec Corporation, GL240) under various applied voltages. For the non-contact measurements, an IR thermal imaging camera (Seek Thermal, Seek Compact, CA, USA) was employed to capture the spatial temperature distribution across the Ag mesh surface. The IR camera enabled the visualization of the heating uniformity and transient thermal behavior without physical contact. All measurements were performed under ambient atmospheric conditions.

## 3. Results and Discussion

Figure S1 shows the OM image of the reference specimen prepared in this study—namely the Ag mesh structure directly printed on the PES film substrate (Ag mesh/PES film). As is evident from the figure, the Ag mesh with rhombic openings, having a line width of ~180 μm and line spacing of ~5500 μm, was uniformly printed across the entire substrate.

**Figure 1:**
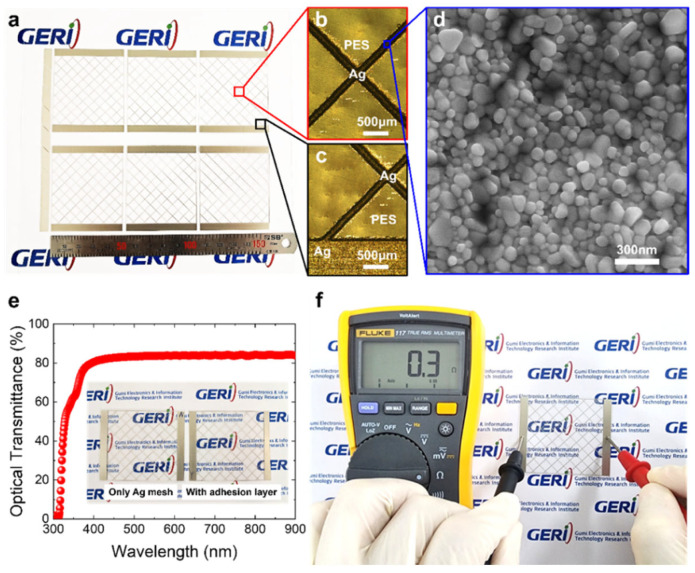
(**a**) Photograph of the Ag mesh electrodes inkjet-printed on PES substrates, with both the adhesion layer and Ag mesh patterned using a piezo drop-on-demand method. (**b**,**c**) OM images of the Ag mesh inkjet-printed on PES: (**b**) center and (**c**) edge of the same sample, showing uniform pattern formation across the entire area. (**d**) FE-SEM image of the inkjet-printed Ag mesh surface, showing densely packed Ag nanoparticles with an average diameter of ~100 nm. (**e**) Optical transmittance spectrum of the printed Ag mesh-based electrode with the adhesion layer, demonstrating high transparency. The inset image shows negligible differences in transmittance with and without the adhesion layer. (**f**) Measured line resistance of the printed Ag mesh-based transparent electrode with the adhesion layer, confirming its excellent electrical conductivity with a low resistance of 0.3 Ω.

Figure 1 shows the structural, optical, and electrical characteristics of the double-layer structure comprising an Ag mesh and dielectric material mesh printed on a PES film substrate (Ag mesh/dielectric material mesh/PES film).

As shown in Figure 1a, six specimens of Ag mesh (thickness: 25 μm)/dielectric material mesh (line width and spacing same as Ag mesh, thickness: 5 μm) of thickness 30 μm and dimensions 50 (width) × 50 mm (length) were successfully printed on the PES film substrate within 60 min, consuming 1.2 mL of conductive Ag ink and 0.3 mL of dielectric ink. Figure 1b–d show surface images of the Ag mesh/dielectric material mesh/PES film observed using OM and FE-SEM. It is evident that the Ag particles, with diameters of approximately 100 nm, were well-assembled into the mesh structure, and no difference in printing quality was evident between the center and the edge of the specimens. These results were consistently observed on both the PI film and glass substrates, as shown in Figures S2 and S3.

Figure 1e shows the optical transmittance spectrum of the Ag mesh/dielectric material mesh structure printed on the PES film. As is evident from the graph, the Ag mesh/dielectric material mesh/PES film exhibited a transmittance of 80–84% in the visible range (400–800 nm). As shown in the inset of Figure 1e, the appearance of the Ag mesh/dielectric material mesh structure (right) was almost indistinguishable from that of the reference specimen—that is, the Ag mesh structure (left). This was also confirmed by the transmittance spectra measurements (Figure S4 and Table S1).

Figure 1f shows the measured line resistance of the Ag mesh/dielectric material mesh/PES film. As is evident from the figure, the line resistance measured between the two ends of the Ag mesh/dielectric material mesh structure, with lateral dimensions of 50 × 50 mm, was 0.3 Ω. The same resistance value was also obtained from the reference specimen without the dielectric material mesh layer.

Figure S5 illustrates the operation of a red light-emitting diode (LED) powered through the Ag mesh/dielectric material mesh structure printed on the PES substrate, confirming its functionality as an electrode. From these experimental results, we demonstrated that the proposed Ag mesh/dielectric material mesh/PES film could be effectively employed as a transparent electrode [28,29,30,31].

**Figure 2:**
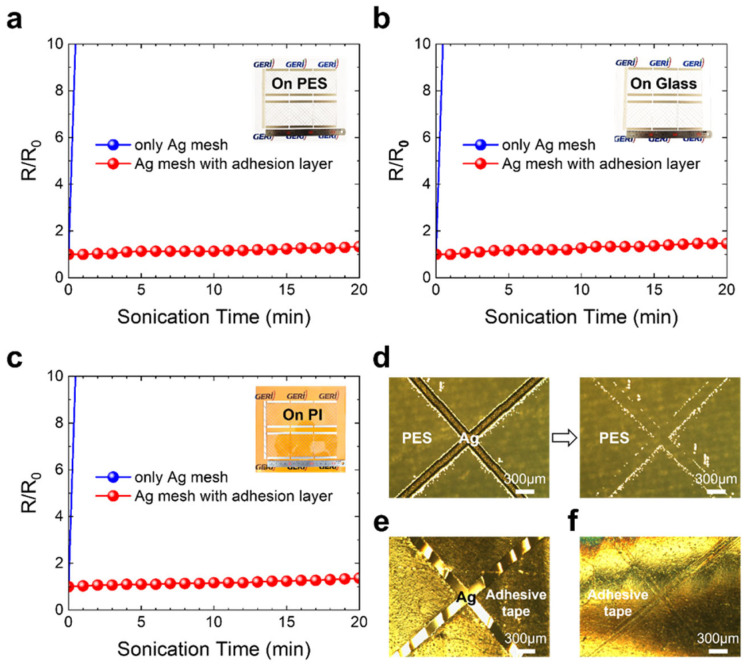
Adhesion test results of the Ag mesh structures printed on the PES film, glass, and PI film substrates with and without the dielectric material as an adhesion layer. (**a**–**c**) Ultrasonic treatment test results. (**d**) Adhesion test results of Ag mesh structures printed directly on the PES substrate using cellophane adhesive tape. (**e**) Ag mesh structures transferred onto the tape after the adhesion test for specimens without the adhesion layer. (**f**) OM image of the tape surface after the adhesion test for specimens with the adhesion layer, showing only faint traces of the mesh structure.

Figure 2 shows the adhesion test results of the Ag mesh structures printed on PES film, glass, and PI film substrates with and without the dielectric material adopted as an adhesion layer.

As is evident from Figure 2a–c, most of the Ag mesh structures printed on the PES film, glass, and PI film without the adhesion layer were delaminated from the substrates during the early stages of ultrasonic treatment, leading to a pronounced increase in resistance of the printed electrode. By contrast, for the specimens incorporating the dielectric material as an adhesion layer, no delamination of the Ag mesh structures was evident even after 20 min of ultrasonic treatment, and no major change in electrical resistance was detected.

Figure 2d presents the adhesion test results using cellophane adhesive tape for the Ag mesh structures printed directly on the PES substrate. As is evident from the figure, the Ag mesh electrodes printed without the adhesion layer were completely detached from the PES substrate after only a single tape test. Moreover, Figure 2e shows that the Ag mesh structures were transferred onto the cellophane adhesive tape after the adhesion test. In sharp contrast, the specimens with the adhesion layer exhibited no evidence of delamination of the Ag mesh structures from the substrates after the tape test.

Figure 2f displays the OM image of the cellophane adhesive tape surface after the adhesion test of the specimen with the adhesion layer, where only faint traces of the mesh structure were evident. Based on these results, we confirmed that the dielectric material could effectively improve the adhesion between the Ag printed electrodes and various substrates (PES film, glass, and PI film) without compromising their electrical or optical properties.

**Figure 3:**
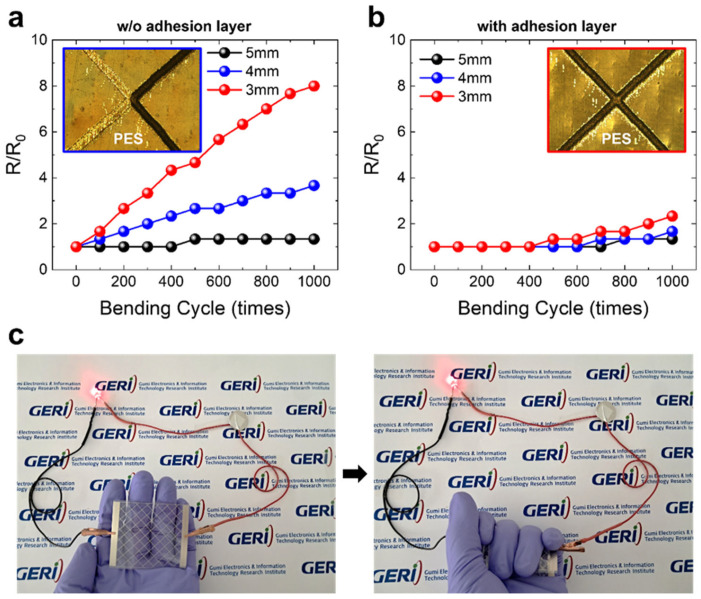
Outer bending test results of the printed (**a**) Ag mesh electrode without an adhesion layer and (**b**) the Ag mesh/dielectric material mesh electrode with an adhesion layer. (**c**) Demonstration of a red LED driven by the printed Ag mesh/dielectric material mesh electrode, showing stable operation even in a crumpled state.

Beyond adhesion, mechanical flexibility is a critical property for printed electrodes designed for flexible and wearable devices [32]. Insufficient flexibility can lead to structural damage under mechanical stress, thereby limiting long-term reliability. In this study, the mechanical flexibility of the printed Ag mesh electrodes was evaluated using outer and inner bending tests with bending radii of 3, 4, and 5 mm. The results of the outer bending tests are presented in Figure 3a,b, whereas the inner bending test results are shown in Figure S6. Resistance variation was more pronounced under outer bending than under inner bending, and the effect became increasingly significant as the bending radius decreased.

Notably, electrodes printed directly on the substrate without a dielectric adhesion layer exhibited a relatively large increase in resistance. Post-test inspection, as shown in the insets of Figure 3a,b, revealed that this behavior resulted from the partial detachment of the Ag mesh from the substrate in the absence of the adhesion layer. These findings indicate that the dielectric adhesion layer played a crucial role in preventing delamination at the electrode–substrate interface and actively maintained the durability of the printed electrodes by preserving their conductive pathways even under severe bending conditions. Figure 3c shows that the printed transparent electrode based on the Ag mesh/dielectric material mesh structure could reliably operate a red LED, even in a crumpled state.

**Figure 4:**
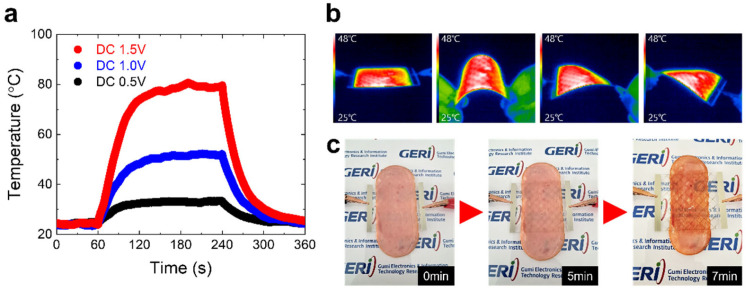
(**a**) Temperature variation of the printed Ag mesh/dielectric material mesh electrode under applied DC voltages 0.5, 1.0, and 1.5 V. (**b**) IR thermal images of the flexible transparent heater during operation. (**c**) Demonstration of the heating performance of the flexible transparent heater under a DC bias of 1.0 V.

Figure 4 presents the experimental demonstration of the proposed printed transparent electrode as a heater. The Ag mesh/dielectric material mesh structure was printed on a heat-resistant PES substrate, and heat generation was induced by applying a voltage across its terminals.

As shown in Figure 4a, the electrode immediately started to generate heat upon voltage application, leading to a rapid increase in temperature, whereas the temperature dropped back to room temperature (~25 °C) once the voltage was removed. The maximum heating temperature increased proportionally with the applied voltage. Figure 4b presents IR thermal images showing that the heating behavior did not deteriorate even when the electrode was bent or twisted under a DC bias of 1.0 V. This robust performance clearly demonstrates that the enhanced adhesion and mechanical flexibility provided by the adhesion layer between the printed electrode and the substrate played a decisive role.

Figure 4c illustrates the heat-generating capability when a pork ham sample of approximate thickness 2 mm was placed on the electrode surface under a DC bias of 1.0 V. The sample gradually heated up, and visible signs of cooking appeared after approximately seven minutes of operation.

## 4. Conclusions

In this study, a mesh-structured dielectric adhesion layer was applied between the substrate and printed Ag mesh electrodes on three types of substrates—that is, PES film, PI film, and glass—to enhance interfacial adhesion and mechanical flexibility. Despite the addition of the adhesion layer, flexible transparent electrodes were successfully fabricated without any loss in optical transmittance. The fabricated electrodes maintained stable electrical performance under various mechanical deformations—including bending, crumpling, and twisting. Moreover, by applying a voltage across the ends of the Ag mesh electrodes, the electrodes functioned as flexible transparent heaters. These results highlight the potential of mechanically robust and transparent electrodes/heaters for integration into next-generation wearable systems, on-device AI platforms, humanoid robots, and ocean exploration equipment, and provide a practical pathway for the scalable implementation of multifunctional flexible/wearable electronic devices [33,34,35,36,37,38].

## Data Availability

The data that support the findings of this study are available from the corresponding author upon reasonable request.

## Supplementary Materials

The following supporting information can be downloaded at: https://www.mdpi.com/article/10.3390/nano15211681/s1, Figure S1: Photographic and OM images of the Ag mesh electrodes inkjet-printed on PES substrates without an adhesion layer. The printed patterns exhibit good geometrical fidelity and uniformity, comparable to those with the adhesion layer shown in Figure 1, indicating that the adhesion layer does not affect the printing resolution or shape definition; Figure S2: Photographic and OM images of the Ag mesh electrodes inkjet-printed on glass substrates without (top) and with (bottom) an adhesion layer, showing consistent pattern formation under both conditions; Figure S3: Photographic and OM images of the Ag mesh electrodes inkjet-printed on PI substrates without (top) and with (bottom) an adhesion layer, showing consistent pattern formation under both conditions; Figure S4: Optical transmittance spectra of the Ag mesh electrodes with (red) and without (blue) an adhesion layer. Both samples exhibit similar transmittance profiles, indicating that the adhesion layer does not significantly affect their optical transparency; Figure S5: Demonstration of the red LED operation using the printed Ag mesh-based electrode, confirming its applicability as a transparent conducting electrode; Figure S6: Inner bending test results of the printed (a) Ag mesh electrode without an adhesion layer and (b) the Ag mesh/dielectric material mesh electrode with an adhesion layer; Table S1: Optical transmittance values of the Ag mesh-based electrodes with and without an adhesion layer, shown as a function of wavelength, corresponding to the data in Figure S4.

## Abbreviations

The following abbreviations are used in this manuscript:

DI	Deionized
FE-SEM	Field-emission scanning electron microscopy
IR	Infrared
LED	Light-emitting diode
OM	Optical microscopy
PES	Polyethersulfone
PI	Polyimide
UV	Ultraviolet
