# Supplementary material for: Printed Ag Mesh Electrodes with Enhanced Adhesion on Diverse Substrates for Transparent Heater Applications
Source: Nanomaterials (Basel). 2025 Nov 5;15(21):1681. doi: 10.3390/nano15211681 (PMC12609140; doi:10.3390/nano15211681)
Supplement: Supplementary file 1 [file nanomaterials-15-01681-s001.zip › nanomaterials-3947011-supplementary.pdf]

nanomaterials

MDPI

SUPPLEMENTARY MATERIALS

Printed Ag Mesh Electrodes with Enhanced Adhesion on Diverse Substrates for Transparent Heater Applications

Han-Jung Kim [1,†], Se Yong Park [1,†], Jeongmin Park [1], Yohan Ko [1], Changjoo Shin [2], Dong-woo Man [2,*] and Yoonkap Kim [1,*]

[1] IT Materials & Components Research Center, Gumi Electronics & Information Technology Research Institute (GERI), Cheomdangieop1-ro 17, Sandong-eup, Gumi, Gyeongbuk, Korea.
[2] Maritime ICT & Mobility Research Department, Korea Institute of Ocean Science & Technology (KIOST), 385 Haeyang-ro, Yeongdo-gu, Busan, Korea.
* Correspondence: mandongw@kiost.ac.kr (D.-W. Man), yoonkap@geri.re.kr (Y. Kim)
† These authors contributed equally to this work.

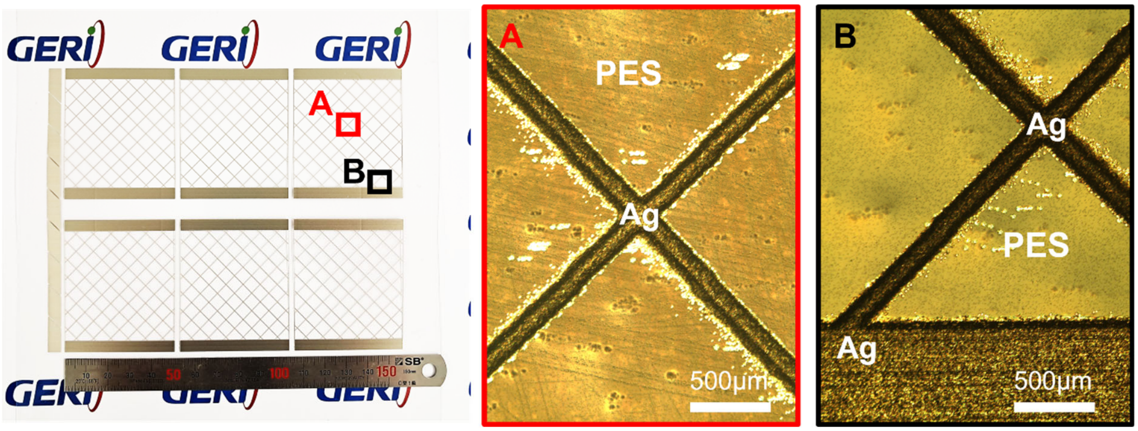

Figure S1. Photographic and OM images of the Ag mesh electrodes inkjet-printed on PES substrates without an adhesion layer. The printed patterns exhibit good geometrical fidelity and uniformity, comparable to those with the adhesion layer shown in Figure 1, indicating that the adhesion layer does not affect the printing resolution or shape definition.

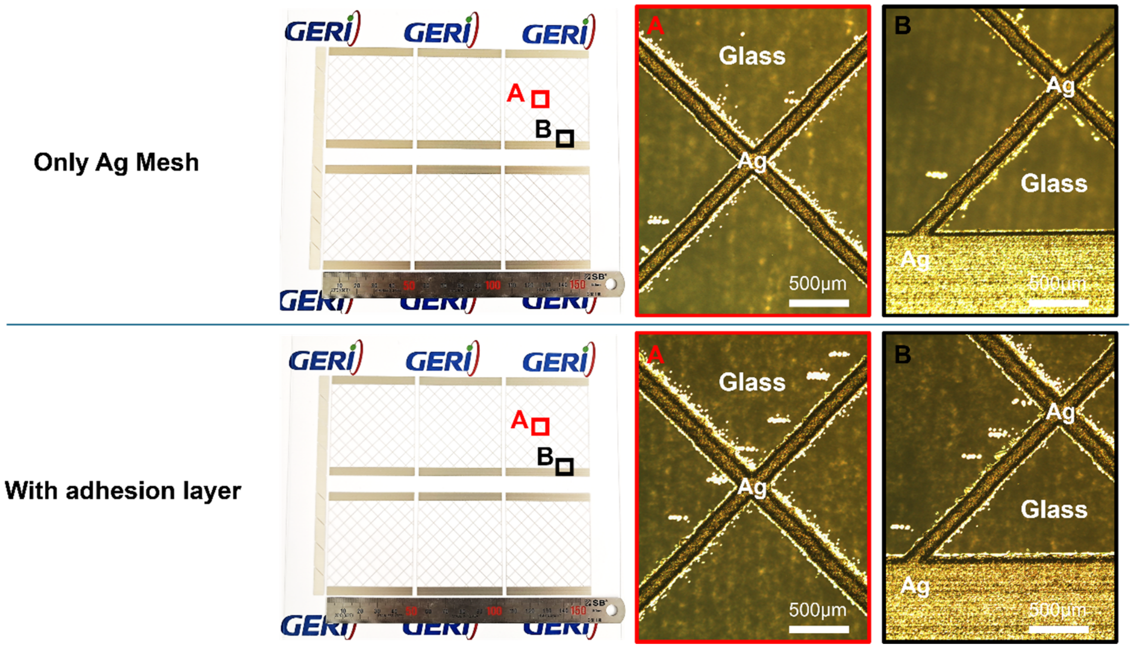

Figure S2. Photographic and OM images of the Ag mesh electrodes inkjet-printed on glass substrates without (top) and with (bottom) an adhesion layer, showing consistent pattern formation under both conditions.

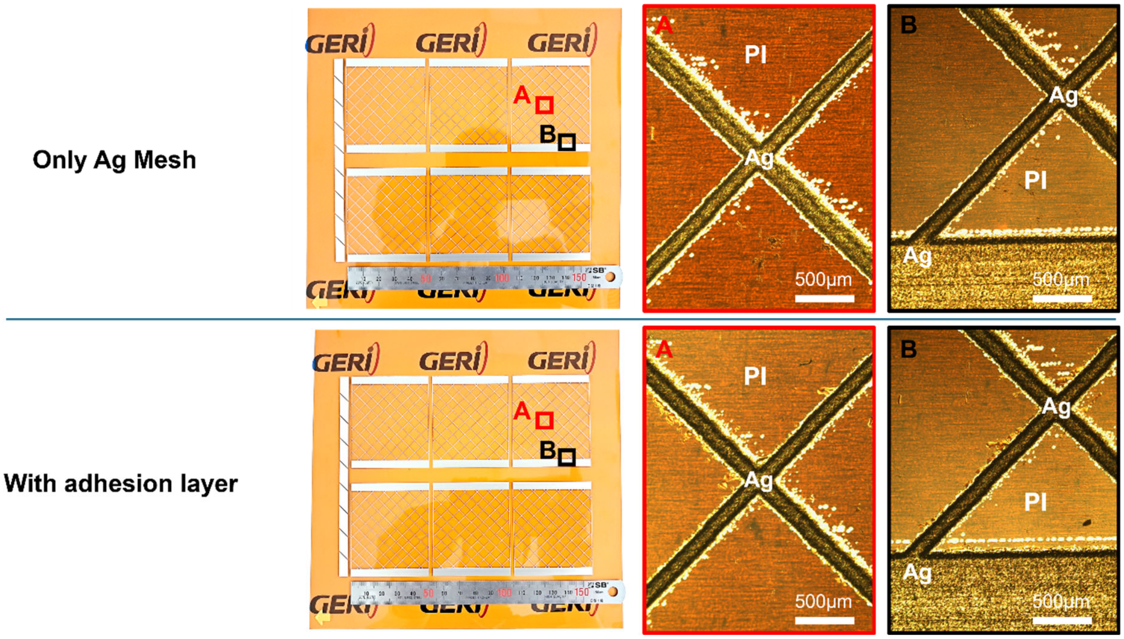

Figure S3. Photographic and OM images of the Ag mesh electrodes inkjet-printed on PI substrates without (top) and with (bottom) an adhesion layer, showing consistent pattern formation under both conditions.

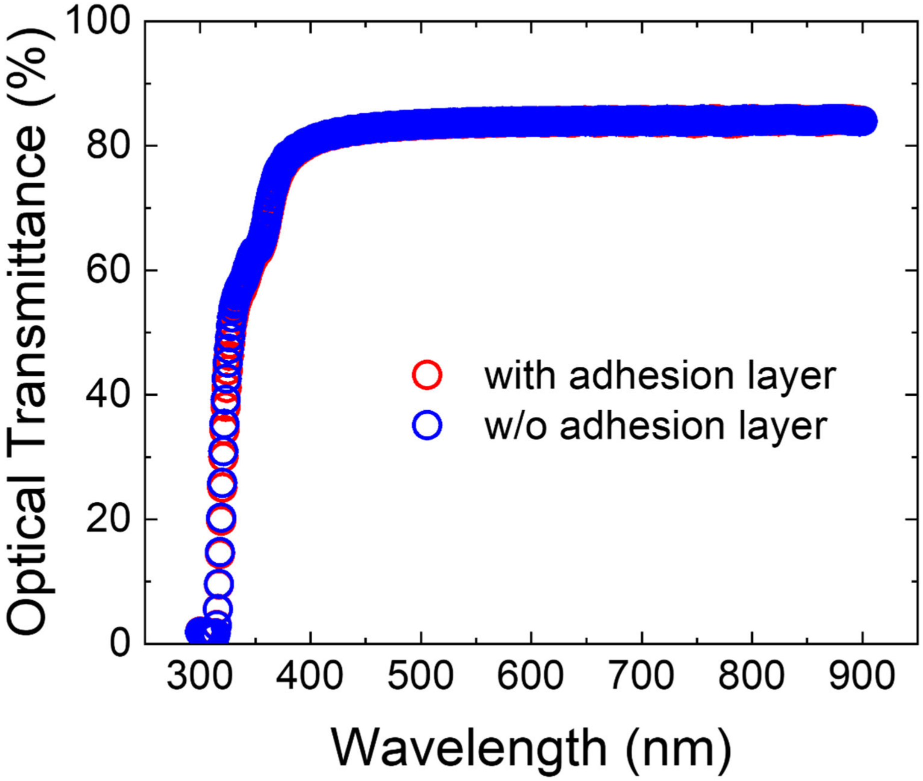

Figure S4. Optical transmittance spectra of the Ag mesh electrodes with (red) and without (blue) an adhesion layer. Both samples exhibit similar transmittance profiles, indicating that the adhesion layer does not significantly affect their optical transparency.

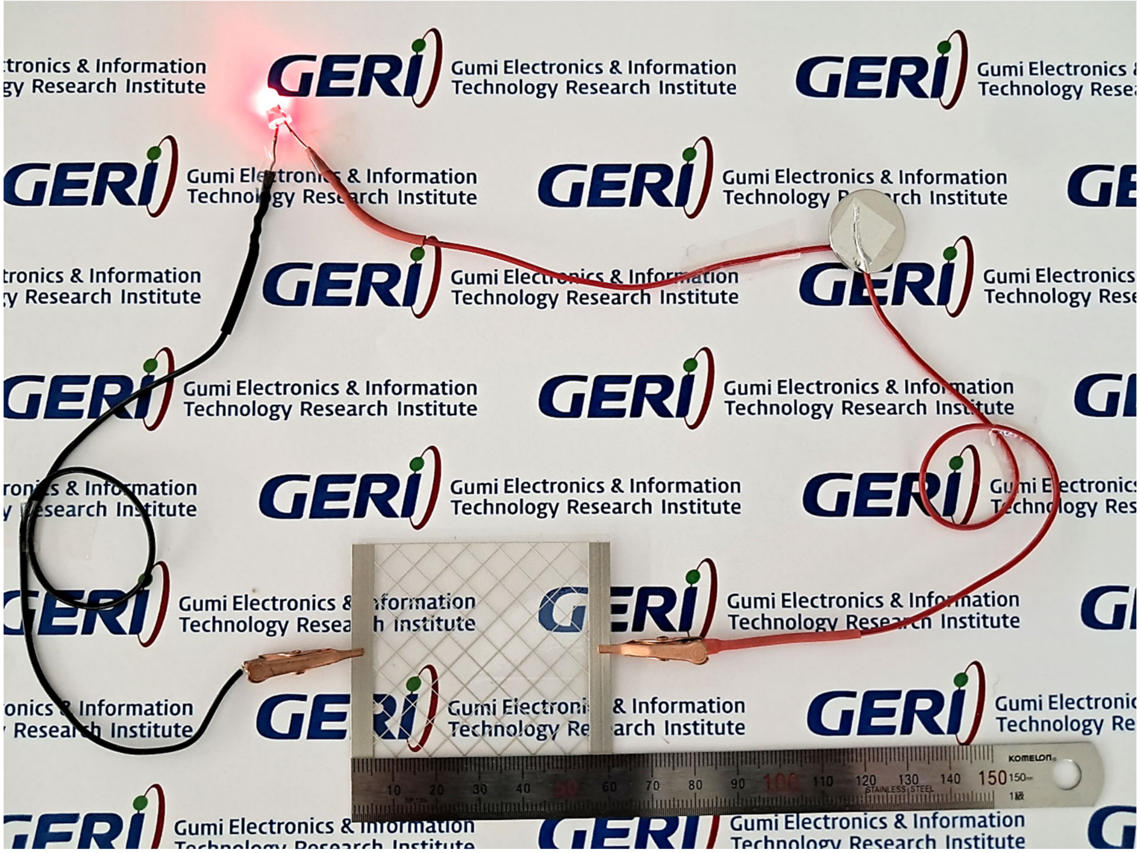

Figure S5. Demonstration of the red LED operation using the printed Ag mesh-based electrode, confirming its applicability as a transparent conducting electrode.

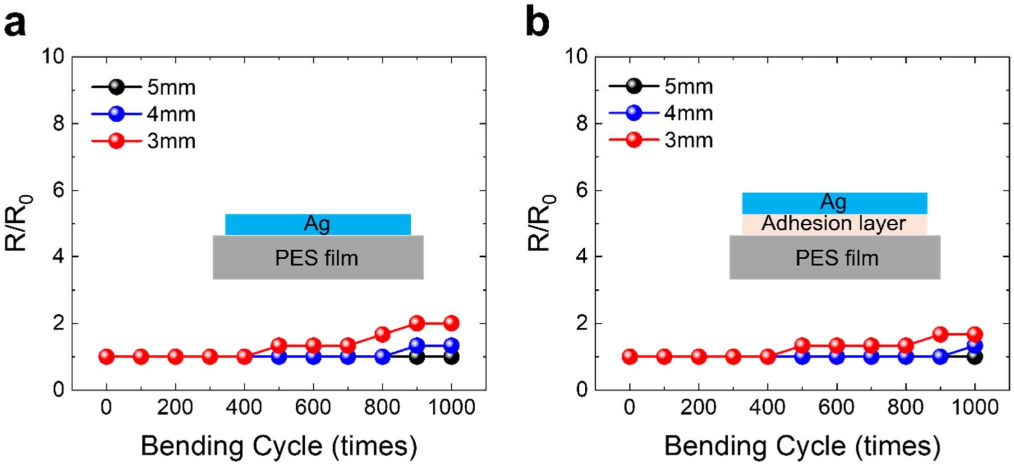

Figure S6. Inner bending test results of the printed (a) Ag mesh electrode without an adhesion layer and (b) the Ag mesh/dielectric material mesh electrode with an adhesion layer.

Table S1. Optical transmittance values of the Ag mesh-based electrodes with and without an adhesion layer, shown as a function of wavelength, corresponding to the data in Figure S4.

Wavelength	Optical Transmittance (nm)	
	**With adhesion layer**	Only Ag mesh
300nm	**1.94**	1.88
350nm	**62.42**	63.04
400nm	**80.47**	80.61
450nm	**82.70**	82.70
500nm	**83.42**	83.44
550nm	**83.66**	83.74
600nm	**83.90**	83.91
650nm	**84.00**	83.94
700nm	**84.11**	84.00
750nm	**83.75**	84.09
800nm	**84.17**	84.14
850nm	**84.04**	83.94
900nm	**83.97**	83.94
